# Long-Term Follow-Up of Tamoxifen Treatment and the Use of Imaging in Psammocarcinoma: A Case Report, Review of the Literature and Discussion of Diagnostic and Therapeutic Challenges

**DOI:** 10.3390/curroncol30120747

**Published:** 2023-11-30

**Authors:** Ida Gideonsson, Pernilla Israelsson, Sara N. Strandberg, Ulrika Ottander

**Affiliations:** 1 Center of Obstetrics and Gynecology, Umeå University Hospital, 901 85 Umeå, Sweden; ida.gideonsson@regionvasterbotten.se; 2 Department of Clinical Sciences, Obstetrics and Gynecology, Umeå University, 901 85 Umeå, Sweden; ulrika.ottander@umu.se; 3 Department of Radiation Sciences, Oncology, Umeå University, 901 85 Umeå, Sweden; 4 Department of Radiation Sciences, Diagnostic Radiology, Umeå University, 901 85 Umeå, Sweden; sara.strandberg@umu.se

**Keywords:** case report, psammocarcinoma, psammoma bodies, ovarian cancer, serous carcinoma, imaging, MRI, FDG PET, CT, tamoxifen

## Abstract

Psammocarcinoma (PsC) represents a rare form of low-grade serous tumor of the ovary or peritoneum. Although ovarian cancer generally has a poor prognosis in its late stages, PsC seems to have a more indolent course. We present a patient with a history of unspecific abdominal pain for more than a year, with sudden acute onset of severe inguinal pain. On admission to the hospital, a computed tomography (CT) revealed a pelvic mass of suspected ovarian origin. Radical surgery was attempted but not achieved due to widespread tumor growth. Histopathological evaluation revealed estrogen receptor-positive stage III PsC. Tamoxifen treatment was thus initiated, still maintaining stable disease 10 years later. The patient has undergone extensive radiological work-up, including CT, chest X-ray, 18F-fluoro-deoxy-glucose positron emission tomography (PET)/CT, 99mTc- hydroxymethylene diphosphonate (HDP) bone scintigraphy, 18F-fluoro-thymidine (FLT) PET/CT, Tc-99m depreotide scintigraphy and magnetic resonance imaging. In conclusion, we demonstrate that PsC has characteristic radiological features and different imaging modalities can be suitable in different clinical situations. In contrast to most other ovarian cancers, PsC does not always warrant adjuvant chemotherapy, even in advanced stages. This emphasizes the need for a deeper knowledge of the biological behavior of this rare tumor, to select the optimal treatment strategy.

## 1. Introduction

Psammocarcinoma (PsC) is a rare serous tumor form that arises from the ovary or peritoneum. Histopathologically, it is associated with psammoma body formation and low-grade atypia. Psammoma bodies are extracellular, thin, evenly distributed calcified spherules with concentric laminations. They are composed of hydroxyapatite deposits on the outer layer of single necrotic, degenerated cells which progressively form a lamellated appearance [1]. They are often found in serous tumors of the ovary, papillary carcinomas of the thyroid gland, and meningiomas, but also in endosalpingiosis, benign tumors, borderline tumors, and nonneoplastic serous epithelial proliferations [2]. Patients with PsC often remain asymptomatic during local disease progression and seek medical advice due to diffuse symptoms related to the intraabdominal spread of the tumor, such as abdominal discomfort and increasing abdominal girth. Extra-ovarian spread and peritoneal seeding are often present at diagnosis [2,3,4,5]. Peritoneal lesions demand a thorough histopathological evaluation since they can be composed of either endosalpingiosis, peritoneal serous borderline tumors (SBT), low-grade serous carcinomas (LGSC), or high-grade serous carcinomas (HGSC). The specific histological diagnostic criteria for ovarian PsC are well defined [2], and have been modified [6] to include primary peritoneal PsC. PsC is described in the literature as a low-grade disease with a more favorable prognosis than other, more common types of serous adenocarcinomas [2,4,7,8,9,10,11,12,13]. Peritoneal PsC has been compared to peritoneal LGSC [14], and the better prognosis seen in PsC patients may be related to the massive overgrowth of epithelial cells as represented by the calcification, resulting in a shortage of viable tumor cells. Still, several case reports describe a more aggressive clinical presentation [15,16,17,18]. Since PsC is a very rare disease, no standardized treatment guidelines exist. Based on case reports, treatment generally consists of cytoreductive surgery [8,17,19,20]. In more advanced cases, or in cases with residual disease, an individual assessment regarding systemic treatment must be made.

We present a case of advanced-stage PsC, presenting with diffuse abdominal symptoms, subjected to extensive radiological investigations and, due to widespread disease, non-radical surgery. Histopathological examination revealed estrogen receptor-positive PsC and the patient was subsequently treated with tamoxifen, maintaining stable disease more than a decade later. During the to date 13 years of follow-up, the patient underwent extensive radiological work-up. This collection of imaging modalities is presented to demonstrate the utility of imaging at different stages of PsC. Magnetic resonance imaging (MRI) is expected to be useful for preoperative monitoring, and with time, and increasing calcifications, computed tomography (CT) will become a more suitable imaging modality. Our case also illustrates for the first time that an estrogen receptor-positive, inoperable, advanced-stage PsC can be successfully treated with tamoxifen alone.

## 2. Detailed Case Description

**Figure 1:**
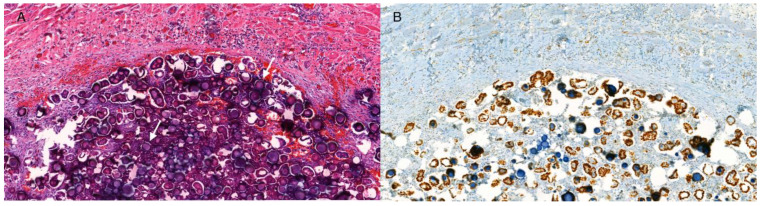
Microphotographs of the tumor (20×). (**A**) Section of the tumor illustrating the abundant psammoma body formation, exemplified by white arrows (hematoxylin-eosin). (**B**) Immunohistochemical estrogen receptor staining.

A 56-year-old woman was admitted to a University Hospital with periodic lower abdominal pain for more than a year, with exaggerated inguinal pain during the last few months. The patient’s medical history was significant for endometriosis, diagnosed with laparoscopy, and later a supra-vaginal hysterectomy due to uterine fibroids and heavy menstrual bleeding. Her body mass index was 23, and she had been on hormonal replacement therapy for three years (1 mg estradiol). A gynecological exam, including vaginal ultrasound, was performed without pathological findings, and no further investigations were conducted. Four months later she was admitted to the emergency ward for severe pain in the right inguinal area and a hernia was suspected. CT of the abdomen showed a 55 × 40 mm multi-cystic, calcified tumor in the right ovary, a herniated partially calcified soft tissue mass in the right groin with a tumor of partially calcified soft tissue, and small calcified nodules in both the Douglas pouch and the greater omentum. At first, tuberculosis or some form of teratoma was suspected. Chest x-ray was normal and Mantoux test was negative. An 18F-fluoro-deoxy-glucose (FDG) positron emission tomography/CT (PET/CT) was performed and there was slight FDG uptake in the herniated soft tissue mass, supposedly a lymph node, at the site of the hernia but no uptake in the ovary or the peritoneal nodules. Tc-99m-HDP bone scintigraphy showed no extraosseous pathological uptake. Tumor markers CA125, HE4, and CEA were within normal limits. A laparotomy with bilateral salpingo-oophorectomy, omentectomy, and a right-side pelvic lymphadenectomy was performed. Extensive adhesions were seen between the greater omentum and the abdominal wall, and between the sigmoid colon and the urinary bladder. The right ovary was visualized, along with the cystic change, and the left ovary was normal. Nodular lesions were found on the anterior aspect of the bladder and on the mesentery. The tumor was not optimally debulked, leaving 15–20 residual nodular lesions <0.5 cm. The histopathologic evaluation showed serous epithelia with low-grade atypia and multiple psammoma bodies (Figure 1A) invading the omentum, the peritoneal biopsies, and a lymph node within the hernia in the right groin. The left ovary was normal in size, containing a small cyst with mild atypia. The right ovary was 55 × 45 × 35 mm in size, also containing a small cyst with serous epithelium with mild atypia and psammoma bodies in the cyst wall. On the outside of the ovary, with no infiltration in the stroma, there were glandular formations with serous epithelium with mild atypia but with an abundance of psammoma bodies. Cytopathologic examination of the abdominal wash demonstrated adenocarcinoma cell groups in papillary configurations with an abundance of psammoma bodies. Immunohistochemical analyses showed strong (more than 80%) estrogen receptor positivity (Figure 1B). The diagnosis was established as PsC originating from the peritoneum, and minimal SBT in both the left and right ovaries. The patient was assigned to the International Federation of Gynecology and Obstetrics (FIGO), stage IIIC.

**Figure 2:**
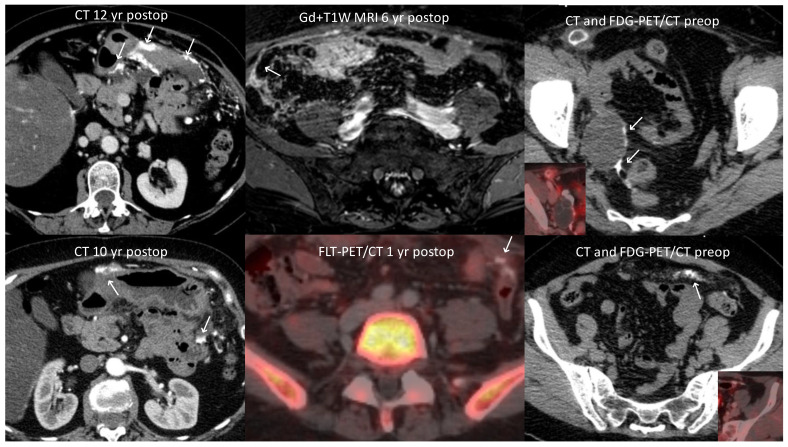
CT and FDG PET/CT at the time of diagnosis (right upper and lower rows) showing a calcified tumor in the right ovary (arrows), a calcified enlarged lymph node in the right inguinal region, and calcified carcinomatosis in the left lower abdominal quadrant (arrow). FLT PET/CT one year after primary surgery (middle lower row) showed no pathological uptake but still visible calcified carcinomatosis in the left lower abdominal quadrant (arrow). Six years after the initial presentation and numerous negative CT and MRI scans, pathological contrast enhancement in the peritoneum in the right fossa (arrow) was detected on MRI (middle upper row). Subsequent MRI scans were negative until a CT was performed for another indication (left lower row), revealing calcified carcinomatosis also in the upper abdomen, particularly adjacent to the ventricle (arrows). The last follow-up CT scan performed 13 years after diagnosis (left upper row) showed further progression of calcified carcinomatosis (arrows). Abbreviations: CT = computed tomography, FDG PET = 18F-fluoro-deoxy-glucose (FDG) positron emission tomography, FLT = 18F-fluoro-thymidine, MRI = magnetic resonance imaging.

**Figure 3:**
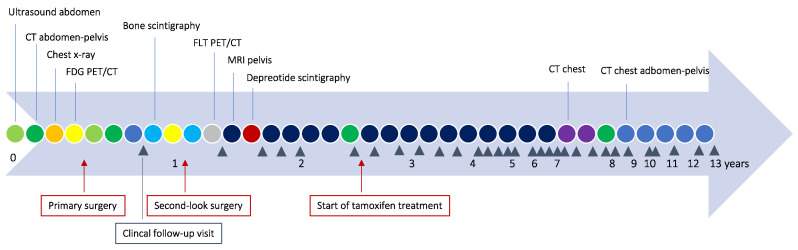
Timeline showing the different imaging techniques, treatments, and clinical follow-up visits. Abbreviations: CT = computed tomography, FDG PET = 18F-fluoro-deoxy-glucose (FDG) positron emission tomography, FLT = 18F-fluoro-thymidine, MRI = magnetic resonance imaging.

Following primary surgery, the patient did not receive post-operative systemic chemotherapy but was admitted to a national center for peritonectomy and hyperthermic intraperitoneal chemoperfusion (HIPEC). Second-look surgery, performed almost one year after primary surgery, showed disease progression with widespread carcinomatosis on the small intestine and an extensive part of the mesentery. Consequently, resection of all gross tumor was deemed impossible. No HIPEC was performed, and only peritoneal biopsies were taken. No radiological method, neither FDG PET/CT nor CT, had shown the extent of disease spread in the abdomen at the time of presentation, and before second-look surgery, a repeated series of FDG PET/CT, and Tc-99m-HDP bone scintigraphy examinations did not show any increased metabolism or pathological osteoblast activity. After second-look surgery, an 18F-fluoro-thymidine (FLT) PET/CT was performed without any signs of increased proliferative activity. A Tc-99m depreotide scintigraphy showed no somatostatin receptor-positive lesions. Eventually, an MRI offered more information and showed widespread changes around the colon and the small intestine. Medical treatment of the tumor was considered given the advanced FIGO stage of the disease. The patient, at this point, had made a full recovery after surgery and was asymptomatic. Systemic chemotherapy was discussed in terms of expected effects and side effects, but treatment with tamoxifen 20 mg 1 × 2 was chosen since the tumor was estrogen receptor-positive. Follow-up visits, including gynecological exams, were performed every third to sixth month. MRIs were conducted yearly without any radiological signs of progress in the pelvis during the first six years. Since the patient was scanned by CT in the protocol of a health screening study seven years after diagnosis, slight signs of progress in the upper abdomen were revealed in years 7–11. At the 13-year follow-up appointment, which occurred recently, the patient had further discrete CT-detected progress in the upper abdomen, but no clinical signs of progress, and the treatment is still tamoxifen, which is well-tolerated by the patient. Hence, the disease has remained clinically stable on tamoxifen for 10 years. A selected number of representative images from CT, FDG PET/CT, FLT PET/CT, and MRI performed throughout the disease, are illustrated in Figure 2. A timeline of the investigations, treatments, and clinical follow-up visits is shown in Figure 3.

## 3. Method

A review of the literature was conducted. A Medline search was performed using the MeSH terms peritoneum, peritoneal, ovary, ovarian neoplasms, and carcinoma in combination with psammoma or psammocarcinoma. Of 233 hits, 54 were considered relevant. Only articles written in English were considered. To verify the accuracy of the search process, reference lists of the 54 articles were scanned for articles not found in our search. Two relevant references were found with a variant spelling, namely “psammomacarcinoma”. All correspondence and some case reports were excluded due to insufficient information on the clinical cases. Case reports before 1990, the year when Gilks et al. published the histological diagnostic criteria of PsC [2], were not included in the summary of clinical data (Table 1). Blaustein Pathology was used as the background [21].

## 4. Results

The findings of the literature review are summarized in Table 1. The mean age for diagnosis was in the mid-50s, and approximately half of patients were diagnosed with primary ovarian origin, and half with primary peritoneal origin. The disease was often diagnosed at an advanced FIGO stage.

## 5. Discussion

### 5.1. Clinical Characteristics and Pathological Evaluation

The current knowledge of PsC can be summarized into a diagnostic framework composed of several recurrent clinical characteristics, as shown in Table 1. Our patient fits well into this framework, being 56 years old at the time of diagnosis and presenting with stage III disease. Ca125 levels were normal, as they often are in peritoneal PsC [19]. The quantity and extent of calcification formed by psammoma bodies were hypothesized to decrease the production of the Ca125 tumor marker, causing it to be deceptively low at such an advanced stage of disease [31]. The histopathological characteristic of PsC is well defined. Thanks to adequate sampling, invasion was identified, and the tumor could be subclassified with accuracy. Of note, our patient also followed the PsC’s typical favorable prognosis, compared to other subtypes of serous adenocarcinomas.

### 5.2. Radiology

PsC is characterized by typical calcifications, but there is, to our knowledge, no comprehensive radiological PsC guideline. We scanned the literature regarding typical characteristic radiological features. Psammoma bodies are described by two properties, density and distribution, which were first described in 1955 [45]. Typically, the density is less pronounced than in almost any other type of calcification, such as in fibroids, calcified lymph nodes, and calculi. Psammoma bodies have a thin shape and a uniform distribution throughout the neoplastic region and produce slightly more dense shadows than what is seen in ordinary tissue. With multiple calcified lesions in the abdomen, some conditions to be considered in the radiological differential diagnosis are pseudomyxoma peritonei, undifferentiated abdominal malignancies, tuberculosis peritonitis, and mucinous adenocarcinoma of the stomach [46]. Radiological features of PsC in CT have been presented as highly condensed and widespread calcifications [27]. Dong et al. [47] suggested that FDG PET/CT may have an advantage over conventional CT in differentiating malignant calcifications from benign calcifications based on metabolic activity. This theory is supported by previous case reports [15,16]. Unfortunately, in our case, neither FDG PET/CT nor CT showed the full extent of abdominal spread, and no increased metabolic activity could be demonstrated in the peritoneal lesions. Due to the prominent calcifications, PsC can incidentally be detected in bone scintigraphy where extraosseous depositions may be the first sign of this diagnosis. For instance, Radin et al. presented a case of stage IIIC PsC of the ovary, primarily suggested by incidental soft tissue uptake on bone scintigraphy [11]. Ranner et al. presented an unusual case of serous cystadenocarcinoma with calcified peritoneal metastases, which could only be demonstrated as abdominal focal uptake on bone scintigraphy, where the corresponding lesions could not be visualized on CT because of their small size [48]. In our case, repeated Tc-99m-HDP bone scintigraphy before primary surgery and after attempted second-look surgery did not show any pathological extraosseous uptake. In addition, Tc-99m depreotide scintigraphy, as well as FLT PET/CT, were performed before second-look surgery without signs of any pathological somatostatin receptor expression or increased proliferation. In the case presented by Hiromura et al., an MRI showed a solid tumor with heterogenous areas of hypodensity on both the T1- and T2-weighted images [27]. The mass was moderately enhanced by meglumine gadopentetate and had a sandy or coarsely granulated appearance on enhanced T1-weighted images.

In summary, PsC is described with specific radiological characteristics (Table 2), and it seems that different imaging modalities can contribute to the preoperative diagnostic process as well as to follow-up. Imaging can be tailored to each stage of disease, taking the strengths and weaknesses of each modality into account. Hence, throughout the diagnostic process and the clinical follow-up, our patient was subject to a discussion about what imaging modality was best suited to her case. CT, FDG PET/CT, FLT PET/CT, Tc-99m-HDP bone scintigraphy, Tc-99m depreotide scintigraphy, and MRI were all utilized (Figure 2), according to decisions made in clinical multidisciplinary conferences. In our case, MRI was key for visualizing disease spread early in the course of the disease and should be considered preoperatively. Considering the long course of PsC with increasing calcification of lesions, follow-up with CT is likely to gradually become the most suitable imaging modality.

### 5.3. Therapy

Given its rarity, there is no established standard therapy for patients with PsC. All the relevant literature on PsC currently consists of case reports or case studies. To our knowledge, there are only four published cross-case studies [2,14,20,32]. Taking these studies into account, the standard treatment is considered maximal surgical debulking, including bilateral salpingo-oophorectomy, total abdominal hysterectomy, and omentectomy [8,17,19,20]. Delhorme et al. presented 25 patients with ovarian or peritoneal PsC, all of whom underwent surgery, where 84% had complete cytoreductive surgery, which was associated with better overall survival and disease-free survival. Furthermore, the absence of preoperative chemotherapy was associated with better overall survival. Conservative surgery has been suggested as an acceptable option in young patients [9].

As presented in Table 1, postoperative medical management has differed from chemotherapy (49 of in total 91 patients) to tamoxifen treatment (3/91), letrozole treatment (1/91), radiotherapy (1/91), or observation only (37/91). In the case of residual disease, only four of the reports (presented with individual details for each patient) have a follow-up period of more than a year. In this subgroup, there are two reports where the patient was treated with chemotherapy [16,35]. Takahashi et al. present a case with a complete response to combination therapy after suboptimal cytoreductive surgery [35]. Debbagh et al. presented a case where surgical exploration revealed peritoneal carcinosis and an ovarian mass, although without long-term follow-up [36]. Histopathologic examination showed PsC and the patient was treated with nine cycles of chemotherapy with complete clinical response, normalization of Ca125, and radiological stabilization. However, there are also reported cases in which second-look surgery following chemotherapy could not show any significant clinical response [9].

There are several cases of long-term survival in patients with advanced-stage PsC with no residual disease who did not receive adjuvant therapy. Of the 13 patients with more than a year’s follow-up (presented in Table 1), one was reported dead of disease after 6.5 years [2], and the remaining patients showed no evidence of disease (NED), with a median follow-up of 3 years. In the corresponding group of 14 patients with advanced-stage PsC with no residual disease and a follow-up period longer than 1 year who did receive adjuvant treatment, 11 reported NED with a median follow-up of 5.5 years. One presented a long-term recurrence 10 years after second-look laparotomy, which could be completely resected [38], one a case with recurrence 2.5 years after radical cytoreductive surgery followed by intraperitoneal chemotherapy [32] and one with recurrence after 5 years, treated with chemotherapy with subsequent stable disease for 15 years until further progression [44].

As chemotherapy entails a substantial risk of side effects and most cases of PsC have a favorable prognosis, hormonal treatment offers an attractive option. Estrogen has tumor-promoting effects in some types of ovarian cancers and to oppose this, selective estrogen receptor modulators (SERMSs), as tamoxifen, were developed [49]. The rationale for using tamoxifen in PsC is that, like LGSC, PsC shows a higher estrogen and progesterone receptor expression prevalence than HGSC [50], suggesting that PsC may respond to hormonal treatment. Endocrine therapy in patients with LGSC is currently under evaluation in several clinical trials, since retrospective data have shown very promising results in women with advanced-FIGO-stage LGSC [51]. Multiple mechanisms are likely involved in the pathogenesis of the widespread calcifications in PsC. However, the in vivo studies on guinea pigs that describe how calcifications in the ovary and endometrium and their neoplasms can be induced by various hormones are of particular interest in this aspect [52]. A few case reports describe the use of tamoxifen or letrozole in postoperative management but only one, before this case report, did so with documented follow-ups [8,15]. Molpus et al. presented a case treated primarily with surgery, where all residual implants were less than 5 mm in size [8]. Four years after initial treatment, the patient presented with ascites and elevated Ca125 (but no abdominal/pelvic mass). Relapse was suspected, with subsequent initiation of treatment with 20 mg tamoxifen twice daily. This resulted in the resolution of ascites, normalized Ca125, and no evidence of metastatic disease at a 4-year follow-up. In our case, there was residual disease after surgery with no sign of progression for the first 7 years. The problem with interpreting the effect of the postoperative therapy with tamoxifen is to determine whether it reflects the indolent nature of PsC or an actual effect of the treatment. Taken together, there are no definitive data regarding the efficacy of chemotherapy in PsC, but adjuvant chemotherapy should be considered, especially in a more aggressive course of disease or when maximal debulking is not successful [14,17,18]. Both conservative management and hormonal treatment can be options, especially when the side effects of chemotherapy need to be avoided.

## 6. Conclusions

In the medical investigation of suspected abdominal spread, PsC has typical characteristics, and therefore imaging can be tailored accordingly depending on the clinical situation. In accordance with our case, MRI can also be considered preoperatively to envision disease spread, while with time and increasing calcification of lesions, CT will gradually become more appropriate for follow-up. If possible, the primary treatment should be maximal debulking surgery. Postoperative chemotherapy, hormonal treatment, or observation can be considered, given the often-favorable prognosis of PsC. We here present the first case of PsC, suboptimally debulked, treated with tamoxifen with subsequent stable disease for more than a decade, highlighting the need for further investigations regarding the postoperative treatment of PsC.

## 7. Patient Perspectives

The patient read and approved the case report after appropriate adjustments based on her comments.

## Figures and Tables

**Table 1:** Reported cases of ovarian and peritoneal psammocarcinoma.

Author Year	*n*	Age	Type	FIGO-Stage	Surgery	Res (cm)	m.t.	Follow-Up (Years)
Gilks, 1990 [2]	11	66	o	IIIB	SOE	0	-	LFU
		72	o	IIIA	SOEB	0	-	NED 10.5
		55	o	IIIA	HSOEB	0	Ct	NED 6.5
		36	o	IIIB	HSOEB	0	-	NED 3
		53	o	IIIB	HSOEB + om	0	-	NED 3.6
		53	o	IIIB	HSOEB + om	NS	-	LFU
		76	o	IIIB	SOEB + om	res	-	PFS < 1
		59	o	IIIC	SOE	0	-	DOD 6.5
		55	p	IIIA	HSOEB + om	0	-	NED 10.5
		48	p	IIIB	HSOEB + om	0	-	NED < 1
		58	p	IIIB	HSOEB + om	0	-	NED < 1
Chen, 1994 [6]	2	59	p	IIIA	HSOEB + om	res < 1	-	PFS 1.5
		71	p	IIIB	px (HSOEB prior)	res	Ct	DOD < 1
Kelley, 1995 [13]	1	18	o	IIIC	HSOEB + om + llg + app + bo	0	Ct	NED 3.5
Powell, 1998 [7]	1	59	o	IIIB	HSOEB + om + llg + app	0	-	NED > 1
Molpus, 1998 [8]	1	58	p	IIIC	HSOEB + om	res < 1	Tx	NED 4 R/CR 8
Poggi, 1998 [18]	1	66	p	IIIB/C	SOEB + om (H prior)	0	-	PFS 1.5
Weir, 1998 [14]	7	48	p	III	2 × HSOEB + om	NS	Ct 5/7	4 LFU
		(27–74)			3 × SOEB 1 × px, 1 × om		RT 1/7	3 NED 1.4, 3.8, 8.3
Lehner, 1998 [4]	1	37	p	IIIB	HSOEB + om + llg	0	Ct	NED 2.4
Munkarah, 1999 [9]	2	27	p	IIIB	SOE + llg + om + app	res < 1, 0 2:nd	Ct	NED 15
		59	p	IIIC	NS	0	-	NED 2
Whitcomb, 1999 [10]	1	59	p	IIIC	HSOEB + om	0	-	NED 2
Piura, 2001 [22]	1	67	p	IIIC	HSOEB + om + llg + bo	0	Ct	NED 1.3
Cobellis, 2003 [23]	1	48	o	IIIA	HSOEB + om	0	-	NED 2
Giordano, 2005 [3]	1	66	o	IIIB	HSOEB + om	0	Ct	NED 1
Radin, 2005 [11]	1	60	o	IIIC	px	res	Ct	PFS < 1
Rettenmaier, 2005 [24]	1	70	o	IIC	HSOEB + om	0	-	NED < 1
Vimplis, 2006 [1]	1	63	o	IIIB	HSOEB + om	res < 1	Ct	NED < 1
Bilgin, 2006 [25]	1	46	p	IIIB	HSOEB + om + llg	0	Ct	NED 5.5
Koumoundourou, 2006 [26]	1	83	p	IIIB/C	HSOEB + om + bo	0	-	NED 4.5
Hiromura, 2007 [27]	1	73	o	IIIC	HSOEB + om	res	Ct	NED < 1
Akbulut, 2007 [17]	1	67	p	IIIC	HSOEB + om + llg	0	Ct + RT	NED 5.5, AWD 10
Riboni, 2008 [28]	1	70	p	IIIB	HSOEB + om	0	Ct	NED 3
Pusiol, 2008 [29]	2	56	o	IIIB	HSOEB + om + llg	NS	Ct	NED
		50	o	IIIB	HSOEB + om + llg	0	Ct	NED 10.5
Bodnar, 2009 [16]	1	52		IIIC	HSOEB + om + llg + spleen + app	res < 1	Ct	PFS 0.7, DOD 2.3
Alanbay, 2009 [19]	2	41	p	IIIB	HSOEB + om + llg + app	0	Ct	NED 6
		50	o	IIIC	HSOEB + om + llg + app	NS	Ct	NED < 1
Zhen-Zhong, 2009 [5]	1	42	p	IIIC	HSOEB + om + llg	0	Ct	NED 1
Sanguedolce, 2009 [30]	1	35	o	IA	myomectomy + SOE	0	-	NED < 1
Tiro, 2009 [31]	1	58	o	IV	Px	res	Ct	PFS < 1
Chase, 2009 [15]	1	45	o	IV	SOEB + px (prev H)	res	Tx	PFS < 1
Poujade, 2009 [32]	4	55	p	IIIC	HSOEB + om	0	-	NED 3
		19	o	IIIC	SOEB + om + llg	0	-	NED 2.4
		67	o	IIIC	HSOEB + om + llg + bo	0	Ct	NED 3.75
		45	o	IIIC	HSOEB + om + bo	0	Ct	NED 2.5, AWD 3
Jain, 2009 [33]	1	55	o	IC	HSOEB	0	Ct	NED < 1
Zakkouri, 2010 [34]	1	52	o	IV	px	res	Ct	AWD < 1
Takahashi, 2012 [35]	1	38	p	IIC	HSOEB	res 4	Ct	NED 1.25
Debbagh, 2013 [36]	1	40	o		px	res	Ct	PFS < 1
Grimaldi, 2013 [37]	1	44	p	IA (?)	bo, 2:nd HSOEB + om + llg + app	0	-	NED 2, R, NED 4
Jena, 2015 [12]	1	60	o	IIIB	HSOEB + om	0	-	NED 1
Norese, 2015 [38]	1	44	o	IIIC	HSOEB + om + llg	res > 1, 0 2:nd	Ct+RT	NED 10, R
Toganel, 2015 [39]	1	54	p	IIIC	SOEB + om + bo		-	NED < 1
Arole, 2017 [40]	1	60	p	IIIB	HSOEB + om	0	Ct	NED 1
Delhorme, 2020 [20]	25	53	o+p	24 III/1 IV	84% complete surgery		Ct 14	median 3.5 8 DOD
		(17–78)						8 AWD 7 NED
Fukagawa, 2020 [41]	1	59	o	IIB	HSOEB + om + llg	0	Ct	8 NED
Helal, 2022 [42]	1	74	p	IIIC	HSOEB + om	NS	-	LFU
Kancharla, 2023 [43]	1	49	p	IIIA2	HSOEB + om + bo + px	NS	Letr	NED 0.75
Sapna, 2023 [44]	1	51	p	NS	HSOEB + om + llg	0	Ct	NED 5, R, AWD 15
Our case 2023	1	56	p	IIIC	SOEB + om + llg (H prior)	res < 1	Tx	AWD 13

Abbreviations: *n* = number of reported cases, res = residual disease after surgery, m.t. = medical treatment, o = ovarian, p = peritoneal, SOE = salpingo-oophorectomy, SOEB = SOE bilateral, HSOEB = hysterectomy and SOEB, om = omentectomy, px = biopsy, llg = lymphadenectomy, app = appendectomy, bo = bowel resection, NS = not specified, Ct = chemotherapy, Tx = tamoxifen, RT = radiotherapy, Letr = letrozole, LFU = lost to follow-up, NED = no evidence of disease, PFS = progression-free survival, DOD = dead of disease, R = recurrence, CR = complete response, AWD = alive with disease.

**Table 2:** Radiological characteristics of PsC.

Imaging Modality	Characteristics	Articles
CT	Less pronounced density, uniform distribution of calcification	Hiromura [27]
FDG PET/CT	Increased metabolic activity	Bodnar [16], Dong [47], Chase [15]
Bone scintigraphy	Prominent calcification	Ranner [48], Radin [11]
MRI	Sandy or coarse granulated	Hiromura [27]

## Data Availability

The data presented in this study are available in this article.
